# Expression, Distribution and Role of Aquaporins in Various Rhinologic Conditions

**DOI:** 10.3390/ijms21165853

**Published:** 2020-08-14

**Authors:** Su Young Jung, Dong Choon Park, Sung Su Kim, Seung Geun Yeo

**Affiliations:** 1 Department of Otorhinolaryngology-Head and Neck Surgery, Myongji Hospital, Hanyang University College of Medicine, Goyang 412-270, Korea; monkiwh35@hanmail.net; 2 St. Vincent’s Hospital, The Catholic University of Korea, Suwon 412-270, Korea; park.dongchoon@gmail.com; 3 Medical Research Center for Bioreaction to Reactive Oxygen Species and Biomedical Science Institute, School of Medicine, Graduate School, Kyung Hee University, Seoul 02447, Korea; sgskim@khu.ac.kr; 4 Department of Otorhinolaryngology—Head & Neck Surgery, School of Medicine, Kyung Hee University, Seoul 02447, Korea

**Keywords:** aquaporin, water channel, transmembrane permeability, allergic rhinitis, chronic rhinosinusitis, nasal polyp, olfaction

## Abstract

Aquaporins (AQPs) are water-specific membrane channel proteins that regulate cellular and organismal water homeostasis. The nose, an organ with important respiratory and olfactory functions, is the first organ exposed to external stimuli. Nose-related topics such as allergic rhinitis (AR) and chronic rhinosinusitis (CRS) have been the subject of extensive research. These studies have reported that mechanisms that drive the development of multiple inflammatory diseases that occur in the nose and contribute to the process of olfactory recognition of compounds entering the nasal cavity involve the action of water channels such as AQPs. In this review, we provide a comprehensive overview of the relationship between AQPs and rhinologic conditions, focusing on the current state of knowledge and mechanisms that link AQPs and rhinologic conditions. Key conclusions include the following: (1) Various AQPs are expressed in both nasal mucosa and olfactory mucosa; (2) the expression of AQPs in these tissues is different in inflammatory diseases such as AR or CRS, as compared with that in normal tissues; (3) the expression of AQPs in CRS differs depending on the presence or absence of nasal polyps; and (4) the expression of AQPs in tissues associated with olfaction is different from that in the respiratory epithelium.

## 1. Introduction

Aquaporins (AQPs), also called water channels, are ubiquitous, integral membrane proteins belonging to a larger major family of intrinsic proteins that form pores in the membranes of biological cells and facilitate the intercellular transport of water [1]. AQPs and other members of their family osmotically regulate water flux in various tissues, and some of these proteins mediate the transport of small solutes, including glycerol. AQPs are generally known to be passive transporters of water. In addition to being crucial for water homeostasis, AQPs are involved in physiologically important transport of molecules other than water, regulation of surface expression of other membrane proteins, and cell adhesion, and they also act as signaling factors in cell-volume regulation [2,3]. To date, 13 types of AQPs have been identified, named AQP0 to AQP12. These proteins have been divided into three groups based on their structural and functional characteristics: (1) orthodox AQPs (AQP0, -1, -2, -4, -5, -6, and -8), which are selectively permeable to water; (2) aquaglyceroporins (AQP3, -7, -9, and -10), which are permeable to glycerol, urea, and other small solutes, in addition to water; and (3) S-aquaporins (super- or subcellular AQP, AQP11, and -12), which have peculiar intracellular localizations and functions that are currently under investigation [4,5,6,7]. AQPs, which are involved in the migration of water into various cells, passively transport water across cell membranes, while also preventing the passage of ions and other solutes.

The primary function of most AQPs is to transport water across cell membranes in response to osmotic gradients created by active solute transport. Molecular dynamics simulations suggest that steric factors and electrostatic interactions in the aqueous pore are responsible for the selectivity of AQPs for water [1]. A subset of AQPs, called aquaglyceroporins, also transports glycerol. The pore of aquaglyceroporins is slightly larger in diameter than that of water-selective AQPs and is lined by relatively hydrophobic residues, as compared with the pore of water-selective AQPs. In addition to mediating the transfer of water and glycerol, there is evidence, some of which is controversial, that some AQPs mediate the passage of gases (CO_2_, NH_3_, NO, and O_2_), various small solutes such as H_2_O_2_ and arsenite, and even ions (e.g., Na^+^, K^+^, and Cl^−^). Non-transport functions, such as cell–cell adhesion, membrane polarization, and regulation of interacting proteins, such as ion channels, have also been suggested for some AQPs [2,3,8]. Extensive data indicate that AQPs, acting in their capacity as water channels, not only regulate cell migration but also regulate common events essential for inflammatory responses in various human diseases.

The nose, the primary organ of smell, also functions as part of the body’s respiratory system, providing air for respiration; conditioning the air by filtering, warming, and moistening it; and cleaning itself of foreign debris extracted during inhalation [9,10]. The epithelium of the nasal mucosa is divided into two functional types: respiratory epithelium and olfactory epithelium [11,12,13]. Studies on pathophysiological mechanisms of the nose tend to reflect these functional and histological differences and can be largely classified into those related to diseases associated with respiratory versus olfactory functions of the nose. Representative diseases of the nose as a respiratory organ are allergic rhinitis (AR) and chronic rhinosinusitis (CRS). A shared pathological mechanism for these two diseases is an inflammatory increase in nasal mucosa congestion and secretion, a similarity that forms the basis of continuing studies of water homeostasis in the nasal mucosa. There have also been studies on the role of water channels such as AQPs in the process of absorbing odorants—gaseous compounds that have entered the nasal cavity—during recognition of odors in the olfactory mucosa. However, to the best of our knowledge, there has yet to be a systematic evaluation of the literature on the relationship between AQPs and various rhinologic conditions.

In this review, we address these gaps in our knowledge by summarizing research on the relationship between AQPs and rhinologic conditions, focusing on the mechanisms by which AQPs affects each condition.

## 2. Relationship between AQPs and the Nasal Respiratory Epithelium

### 2.1. Expression of AQPs in Respiratory Epithelium of the Nasal Cavity

The nasal cavity is divided into two segments: the respiratory segment and the olfactory segment. The respiratory segment constitutes the bulk of each nasal cavity and is lined with ciliated pseudostratified columnar epithelium. This type of epithelium is characteristic of all conductive passages dedicated to the respiratory system and is therefore also called respiratory epithelium. Mucus-producing Goblet cells are present in this epithelium. Secretion by Goblet cells is supplemented by mucous and serous glands in connective tissue, underlying the epithelium termed the lamina propria. Veins in the lamina propria form thin-walled cavernous sinusoids, also called cavernous bodies [11]. Epithelial cells of the nasal respiratory tract play an important role in maintaining an equitable temperature by moisturizing the surface, always keeping it slightly lubricated [9,10], highlighting the importance of moisture regulation in the nasal cavity through water/ion channels like AQPs. Expression of AQPs in the respiratory epithelium of the nasal cavity is summarized in Table 1. 

Among the 13 currently known AQPs, AQP1, -2, -3, -4, -5, -7, and -11 are reported to be expressed in the respiratory epithelium of the mammalian nasal cavity [14,15,16,17,18,19,20,21]. AQPs are categorized according to their function and features: AQP1, -2, -4 and -5 are classified as orthodox AQPs; AQP3 and -7 are aquaglyceroporins; and AQP11 belongs to the unorthodox AQP group [4,5,6,7]. Thus, a full range of AQPs subgroups is generally expressed in the respiratory epithelium of the nasal cavity. The presence of these various AQPs forms the basis for predicting that AQPs play a variety of roles in regulating nasal epithelial moisture in the context of respiratory function.

Orthodox AQPs are water channels that play important roles in controlling water permeability in various cells [6,7]. Certain aspects of their functions and distribution are not fully known, but they are present in organs where water homeostasis is crucial, such as the kidney, lung, and brain. 

The AQP1 protein is encoded in humans by the *AQP1* gene. Although distributed in various tissues, its physiological activity is especially pronounced in the kidneys, where it serves as a water channel. AQP1 is a membrane channel that allows rapid water movement driven by a transmembrane osmotic gradient. It also has a secondary function as a cyclic nucleotide-gated ion channel [22]. It has been reported that AQP1 is distributed in various parts of the respiratory epithelium in the nasal cavity. In an immunohistochemical (IHC) localization study in rats, AQP1 was not detected in the surface epithelium of the nasopharynx or conchus, but was abundant in capillaries and venules beneath the nasopharyngeal epithelium, as well as in capillaries and venous sinuses of the nasal conchus [14]. Another study showed that AQP1 is expressed in endothelial cells of blood vessels and surrounding connective tissue cells in the olfactory and respiratory mucosae [15]. The expression of AQP1 mRNA was also confirmed in mice, using quantitative reverse transcription polymerase reaction (qRT-PCR) [16]. In addition, the expression and distribution of AQP1 within a tissue were shown to be similar across different species, including rat, musk shrew, and sheep [17,18]. IHC studies on human inferior turbinate tissue showed that AQP1 is expressed in fibroblasts, especially in the subepithelial area, and in endothelial cells of blood vessels [19]. In another IHC localization study investigating the expression of AQPs in normal human sinonasal mucosa, it was reported that AQP1 is localized to vasculature and connective tissue [20]. Collectively, these observations suggest that AQP1 may be involved in water transfer across the blood vessel wall.

AQP2 forms a channel that carries water molecules across cell membranes. In the kidney, it is found in structures called collecting ducts, where it plays an essential role in maintaining the body’s water balance [23]. One IHC study performed on inferior turbinate tissue from humans reported that AQP2 is localized in the cytoplasm of epithelial cells and acinar cells [19], although these findings were not reported by any other previous studies. Assuming a limited distribution of AQP2 to the collecting duct, the importance of this subtype in the respiratory function of nasal cavity is postulated to be relatively weak, as compared with other AQPs. 

AQP4 is the most common AQP in the brain, spinal cord, and optic nerve. It reaches its highest expression in the human body in the endfeet of astrocytes [24]. AQP4 is also expressed in epithelial cells of many organs throughout the human body, such as the kidney, intestine, salivary glands, sensory organs, and skeletal muscles [25]. In the specific case of expression in epithelial cells, which exhibit apical-basal polarity, AQP4 is concentrated within the basolateral membrane layer [26]. Similar results were observed in studies of the nasal mucosa of rats, mice, and humans [14,15,16,20]. In rats, AQP4 was found in basolateral sites of acinar cells and columnar cells in the epithelium of the nasal mucosa, and it was particularly abundant in basolateral membranes of columnar cells of the nasopharyngeal epithelium [14,15,16]. In contrast, AQP4 was barely detected in nasal cavity tissue of the musk shrew [17]. Hence, the distribution and/or expression of AQP4 may differ depending on the species. In addition to being the primary water channel protein that maintains homeostatic balance within the CNS, AQP4 provides fast water transport [27]. AQP4 may also be involved in various physiological processes, such as waste removal and fine-tuning of potassium homeostasis [28]. Taken together, these observations suggest that such cells could experience rapid increases or decreases in cell volume were it not for their capacity for large transcellular water flow [29]. Alternatively, the presence of AQP4 in the basolateral plasma membranes of respiratory epithelial cells could confer high water permeability on this cellular surface, thereby preventing extensive changes in cell volume during periodic loss of water from the apical plasma membrane through airway humidification during inspiration and expiration. Although changes in expression of AQP4 may be a mechanism for controlling water flux, the rapid translocation of AQP4 in astrocytes is thought to play an important role during the acute phase of cytotoxic edema [30,31]. These results suggest a new hypothesis, that water flux is controlled by the rearrangement of distribution of AQPs not by a change in their expression. Therefore, studies similar to those in astrocytes are necessary to evaluate the surface trafficking/subcellular localization of AQPs on cells in the nasal cavity.

AQP5 is involved in the generation of saliva, tears, and pulmonary secretions [32,33,34]. AQP5 facilitates fluid secretion in submucosal glands, indicating that the luminal membrane of serous epithelial cells is the rate-limiting barrier to water movement [35,36]. The distribution of AQP5 to nasal tissues has been confirmed in rats and mice; in rats, especially, AQP5 is localized to the apical membrane of subepithelial glandular cells in the nasopharynx, but it is absent from the surface epithelium. Moreover, AQP5 is present in the apical plasma membrane of intraepithelial glands of the nasal conchus, but it is absent from subepithelial glands [14,15,16]. The expression of AQP5 on the apical surface of nasal respiratory epithelium has also been confirmed in musk shrews [17]; apical expression of AQP5 has also been shown in glandular acini and ciliated columnar epithelial cells of sheep [18]. In human nasal mucosa, strong localization of AQP5 is observed in apical and basolateral membranes of the surface epithelium, with less intense AQP5 localization in the cytoplasm of these cells. AQP5 is also strongly localized in apical membranes of the glandular epithelium, with lesser localization to the cell cytoplasm and basolateral membrane [20]. There is also evidence that AQP5 is expressed in apical sites of acinar cells and epithelial cells in human nasal epithelium [19]. 

Among the AQPs found in the mammalian nasal cavity are the aquaglyceroporins, AQP3 and AQP7. As noted above, AQP3 and AQP7 are protein channels that can mediate the passage of glycerol, urea, and other small solutes, in addition to water molecules. AQP3 is a water-channel protein that permits rapid and selective water transport across the membrane of the human airway epithelium in response to osmotic gradients [14]. AQP3 is also found in the skin, lungs, cornea, esophagus, colon, stomach, liver, intervertebral discs, and sperm. In addition, it is present in basolateral cell membranes of principal collecting duct cells, where it provides a pathway for the cellular exit of water [37]. In rats, an IHC localization study showed that AQP3 is present in basal cells of tracheal and nasopharyngeal epithelia and is abundant in basolateral membranes of surface epithelial cells of the nasal conchus [14]. In other studies, also in rats, it was reported that AQP3-positive ciliated cells were more evident in the nasal cavity, which is part of the upper reaches of the respiratory tract, than in the trachea or bronchi (lower portion) [15,21]. The expression of AQP3 mRNA has also been confirmed by qRT-PCR in the nasal epithelium of mice [16] and in ciliated columnar epithelial cells and basal cells in sheep [18], but it was largely undetectable in musk shrews [17]. An IHC study on inferior turbinate tissue from humans showed localization of AQP3 at basolateral sites of epithelial cells and acinar cells [19]. An IHC analysis of the expression of AQPs in normal sinonasal mucosa of human found that AQP3 is strongly localized at the basolateral membrane of both surface and glandular epithelium and less strongly at the apical membrane and in the cytoplasm [20]. 

The sequence of AQP7 is closer to the sequences of AQP3 and AQP9 than to those of other AQPs, suggesting that AQP3, -7, and -9 may constitute a subfamily. Members of this subfamily may be important in thermoregulation, including perspiration, and in sperm function [38,39]. AQP7 is also expressed abundantly in both white and brown adipose tissue [40]. AQP7-knockout mice manifest progressive adipocyte fat accumulation and hypertrophy [41], suggesting that downregulation of AQP7 may result in reduced glycerol release from adipocytes. In nasal tissue, an IHC analysis of the expression of AQPs in normal human sinonasal mucosa reported that AQP7 is localized to the cytoplasm of cells of the surface and glandular epithelium [20]. However, no research has clearly reported the role of AQP7 in nasal tissue.

Overall, localization data for AQP3 and AQP7 suggest that these proteins may be involved in the movement of water and other molecules (e.g., glycerol, urea, and other small solutes) between subepithelial connective tissues and epithelial cells [42].

AQP11 is a relatively newly discovered member of the AQP family, and not much is known about its function. AQP11 is expressed in humans and mice, but its specific functions in the nasal respiratory epithelium have not yet been studied [16,20]. 

In summary, abundant expression of AQPs in the respiratory epithelium of the nasal cavity strongly suggests the participation of these proteins in normal physiological processes, such as humidification of inspired air, but also suggests that alterations in their function or expression could contribute to the pathogenesis of nasal congestion and rhinorrhea [13,14,15,16,17,18,19,20,21].

### 2.2. Factors that Influence the Expression of AQPs in the Respiratory Epithelium of the Nasal Cavity

A number of studies have reported on the expression of AQPs in the respiratory epithelium of the nasal cavity; these studies are summarized in Table 2.

Osmotic stress has been reported to regulate the expression of AQPs in nasal respiratory epithelium. For example, when ducks were subjected to an osmotic stress consisting of hypertonic (1% NaCl) solution, levels of expression of AQP1 and AQP5 were reduced by nearly 40% each, as compared with controls [43]. This stress, however, may stimulate only part of the nasal glands, leaving other parts unaffected. Assays in stimulated glands showed that the expression of AQPs was decreased in duct cells and capillaries, which may prevent the dilution of fluid initially secreted in the basal state. In unstimulated cells, however, dilution of secretions through AQP-mediated transcellular water flux was expected to maintain homeostasis. In contrast, another study reported that osmotic water permeability was increased by hyperosmotic stress in human airway epithelium cultured from human nasal polyp tissue, in association with an increase in cellular levels of AQP5 and localization of AQP5 to the apical cell membrane [44]. These findings suggested that hyperosmotic stress may enhance the expression of AQP5, thereby increasing osmotic water permeability. In this scenario, exposure of the nasal respiratory epithelium to hyperosmotic stress might increase water permeability by increasing the expression of AQP5 in apical cell membranes, thereby regulating water homeostasis. Additional studies are needed to determine the reasons that AQP expression patterns differ in response to hyperosmotic stress.

It has also been reported that chitosan promotes the expression of AQP3 and AQP5 [45]. In this study, AQP3 and AQP5 expression was shown to increase in chitosan-treated HNEpCs cultured from the human inferior turbinate, a response that required some time to fully develop. Chitosan, like glycosaminoglycan in the extracellular matrix, has been widely used in tissue engineering, mainly because of its beneficial effects on wound healing through improved hemostasis and antimicrobial activity, and accelerated tissue regeneration. Given recent reports suggesting that chitosan-based biomaterials promote mucosal healing, reduce postoperative adhesion formation, and restore the mucociliary function of sinonasal mucosae, these authors proposed that chitosan increases the expression of AQP3 and AQP5 in HNEpCs by maintaining water balance and regulating periciliary fluid—which is essential for proper mucociliary function [45]—at adequate levels. 

Various signaling pathways have been shown to regulate the expression of AQPs in the nasal respiratory epithelium. For example, the cAMP-PKA/CREB (cyclic adenosine monophosphate-protein kinase A/cyclic adenosine monophosphate response element-binding protein) pathway was shown to be involved in regulating the expression of AQP5 in rat nasal epithelium [46]. CREB is a transcription factor that regulates the expression of genes involved in a number of physiologic functions. Upon phosphorylation at serine-133 (Ser133), CREB binds as a homodimer or heterodimer to the cAMP-response elements (CRE) of many cAMP-responsive genes. CREB phosphorylation status correlates with the stimulation of the transcription of CRE-containing genes, and Ser133-phosphorylated CREB (p-CREB) is a powerful activator of AQP5 expression. An analysis showed that the expression of AQP5 and p-CREB in rat nasal epithelial cells was decreased in treatment with H89, which inhibits cAMP-dependent protein kinase (PKA), but it increased after treatment with forskolin, which enhances cAMP production and PKA activity by stimulating adenylyl cyclase. These findings indicated that AQP5 expression is regulated by the cAMP-PKA/CREB pathway. The mechanisms that regulate the expression of AQP5 in major diseases occurring in the nasal respiratory epithelium were further evaluated. 

Expression of AQP3 and AQP4 in human nasal epithelial cells (HNEpCs) was shown to depend on culture conditions, indicating that AQP3 and AQP4 expression was greater in cells grown on permeable filters than in cells grown on dishes. Because the membrane polarity of epithelial cells is maintained on permeable filters, maintenance of epithelial cell polarity may contribute the increased expression of AQP3 and AQP4 in HNEpCs [47]. The differences observed under different culture conditions may be due to culture-condition-dependent changes in cell polarity, but the exact mechanism remains unknown.

### 2.3. Relationship between AQPs and Allergic Rhinitis 

Allergic rhinitis (AR) is a type of inflammation in the nose that occurs when the immune system overreacts to allergens in the air. AR results from type I hypersensitivity reactions associated with immunoglobulin E (IgE), which mediates allergic responses associated with nasal inflammation of variable intensity. AR affects approximately 10–30% of adults and ~40% of children, and its prevalence is tending to increase [48]. Thus, owing its high prevalence and negative impact on the quality of life, AR is considered a major chronic respiratory disease.

Elevated IgE acts on mast cells to induce the release of histamine, which plays a major role in AR, by promoting allergy and inflammation; it also plays an important role in the secretory response of submucosal glands in the nose. In addition, histamine causes vasodilatation, tissue edema, and sneezing [49]. Another characteristic feature of AR is glandular hypersecretion. A number of studies have clearly demonstrated that AQPs play a substantial role in maintaining fluid balance in airways, such as the nasal cavity. Thus, AQPs are also associated with disordered water metabolism in AR. Studies investigating the relationship between AQPs and AR are summarized in Table 3. 

The best-studied AQP in the context of AR is AQP5, a member of the orthodox AQP subfamily. AQP5 is most likely the primary water channel in the human nasal mucosa, where it acts as a key tight junction protein in the maintenance of mucosal water homeostasis. It is a key molecular player in fluid secretion and a rate-limiting barrier to the secretion observed during allergic inflammation [32,33,34,35,36]. AQP5 is functionally important for regulating airspace-capillary osmotic water permeability. The airways of AQP5-knockout mice are hyper-responsive to cholinergic stimulation, as compared with those of wild-type mice [41]. 

NF-κB, a well-known transcription factor, is a heterodimer composed of the Rel proteins, p50, and p65. NF-κB activity is negatively regulated by inhibitory κB (I-κB), a transcription factor that binds and inhibits NF-κB activation. Together, these two transcription factors are involved in regulating the transcription of a number of immune- and inflammatory-response genes [60]. The factors that stimulate NF-κB include cytokines such as interleukin (IL)-1β and tumor necrosis factor (TNF)-α, protein kinase C activator, viruses, and oxidizing agents like ozone. Activation of NF-κB by such stimulants regulates the expression of genes involved in immune and inflammatory response, such as IL-1β and IL-8, RANTES, and eotaxin [61], which play vital roles in the initiation and perpetuation of allergic inflammation. Thus, NF-κB plays an important role in the regulation of AR cytokine networks [62]. 

In a rat model of AR, AQP5 expression was shown to decrease in the AR group, as compared with the control group, and the decrease that was suppressed by treatment with the NF-κB inhibitor proline dithiocarbamate (PDTC). The expression of p-CREB showed a similar pattern. In contrast, NF-κBp65 and IL-1β were strongly increased in the AR group, an effect that was also suppressed in the PDTC-treated group. These data show that the NF-jB pathway could downregulate AQP5 by IL-1β, which inhibited CREB phosphorylation, or by NF-jBp65, which competitively bound CBP [50]. 

In another AR study by Chang et al., using HNEpCs as a model [51], the NF-κB pathway was reported to suppress the expression of AQP5. Histamine, which induces hypersecretion by the nasal mucosa, is one of the most significant contributors AR pathophysiology. Moreover, CREB, which has been reported to by phosphorylated following histamine stimulation, is an important mediator of histamine effects, playing an important role in regulating histamine action at the gene-expression level. Against this backdrop, these authors investigated the effects of histamine and chlorpheniramine on the expression of AQP5 in HNEpCs and assessed the involvement of the NF-κB pathway in these effects. Following treatment with different concentrations of histamine and/or the antihistamine chlorpheniramine, they analyzed the expression of p-CREB, AQP5, and NF-κB protein by Western blotting. They found that histamine inhibited the expression of AQP5 and p-CREB in HNEpCs, whereas chlorpheniramine caused a concentration-dependent increase in the levels of these proteins. One study investigated the effect of different concentrations of histamine on AQP5 expression in HNEpCs and the underlying mechanism, focusing on p-CREB (Ser133), which increases the expression of AQP5 [52]. Histamine induced a concentration-dependent inhibition of p-CREB (Ser133) in HNEpCs, in association with a concentration-dependent downregulation of AQP5 mRNA and protein. These findings suggested that histamine downregulates AQP5 production in HNEpCs by inhibiting p-CREB (Ser133), which can cause hypersecretion in the AR by impairing clearance of airway epithelial secretions.

Chlorpheniramine had the ability to reverse the inhibitory effects of histamine. H1 antihistamines, such as chlorpheniramine, are among the most frequently prescribed medications for AR. These drugs not only antagonize the immediate allergic reaction, but they also exert inhibitory effects on the expression of adhesion molecules and the release of leukotrienes, inflammatory cytokines or chemokines, thereby inhibiting eosinophil migration and neutrophil infiltration during the late-phase reaction [63]. The study by Chang et al. also demonstrated an additional anti-inflammatory effect of H1 antihistamines through inhibition of CREB phosphorylation. They further showed that cells maintained higher constitutive p-CREB activity in the presence of chlorpheniramine, which caused a substantial concentration-dependent increase in membranous AQP5 expression [51]. 

A number of drugs in addition to antihistamines have been reported to affect NF-κB pathway-dependent expression of AQP5 in an AR model. Li et al. [53] examined whether glycyrrhizin, a triterpenoid saponin that is the main bioactive component of the licorice root extract, affects the expression of AQP5 by virtue of its anti-inflammatory effects. This study showed that expression of AQP5 and p-CREB (Ser133), a powerful regulator of AQP5 transcription, was decreased by inhibition of PKA with H89 and increased by stimulation of PKA with forskolin. On the basis of these findings, the authors concluded that the expression of AQP5 is regulated through cANF-κBp65, an indicator of NF-κB activity that was increased by stimulation with histamine, but it was decreased by glycyrrhizin treatment. They further showed that suppression of inflammation (glycyrrhizin treatment) reversed the effect of histamine on AQP5 expression in HNEpCs, and that the NF-κB pathway was involved in the effect. 

Cholinergic stimulation plays a major role in the pathophysiology of inflammatory airway disease. Parasympathetic nerves provide the dominant autonomic innervation of the airways, and acetylcholine is the predominant parasympathetic neurotransmitter. Acetylcholine activates postjunctional muscarinic receptors present on airway smooth muscle, submucosal glands, and blood vessels, inducing bronchoconstriction, mucus secretion, and vasodilatation [64]. In addition, the parasympathomimetic drug, methacholine, was found to disrupt airway surface liquid homeostasis and showed, using HNEpCs, that this effect was caused by methacholine-induced activation of the NF-κB pathway and subsequent downregulation of AQP5 and p-CREB [54]. Dexamethasone suppressed these effects of methacholine, including NF-κB activation. This latter finding was expected, because dexamethasone, an anti-inflammatory glucocorticoid, exerts its anti-inflammatory activities by modulating the transcription of inflammatory mediators through inactivation of NF-κB and activator protein (AP)-1 signaling cascades.

Many studies have suggested that the mechanism of action of AQP5 in hypersensitivity of the lower airways involves cholinergic stimulation. The significantly increased hyperreactivity to bronchoconstriction observed in AQP-knockout mice was shown not to be due to differences in tracheal smooth muscle contractility in isolated preparations or to altered levels of surfactant protein B. Rather, concentration-dependent bronchoconstriction, in response to intravenously administered acetylcholine, was significantly increased, as shown by an increase in total lung resistance and a decrease in dynamic lung compliance [65]. The *AQP5* gene is located on human chromosome 12q and on mouse chromosome 15, and these genetic loci are associated with airway hyperresponsiveness associated with asthma [65]. These results suggested that the pathway by which AQP5 induces hypersensitivity in response to cholinergic stimulation is related to the location of these genes. Future studies are needed to determine the precise mechanism of action of AQP5.

The type I hypersensitivity reaction, one of the main pathological mechanisms in AR, is characterized by eosinophilic inflammation accompanied by increases in type 2 cytokines, such as IL-4, IL-5, and IL-13, in affected tissues and blood [66]. Numerous studies have shown that IL-13 is a central regulator of T-helper type 2 (Th2)-dominated disorders such as asthma, and some studies have suggested that IL-13 blockade prevents allergen-induced airway inflammation [67]. It was shown that IL-13 is capable of acting directly on epithelial cells rather than through traditional effector pathways involving eosinophils and IgE-mediated events. Accordingly, Skowron-Zwarg et al. [55] investigated the impact of IL-13 on the expression of AQP3, -4, and -5 in HNEpCs. They found that IL-13 did not affect AQP3 or AQP4 expression, but it did abolish AQP5 expression. Since previous studies had reported that IL-13 promotes the expression of TNF-α [68] and TNF-α suppresses the expression of AQP5 [69], the authors proposed that it is possible that IL-13 mediates AQP5 inhibition by activating TNF-α. Ovalbumin (OVA)-induced asthma in AQP3-knockout mice resulted in significantly reduced airway inflammation compared with OVA-induced asthma in wild-type mice. Adoptive transfer experiments showed that airway eosinophilic inflammation was lower in mice receiving OVA-sensitized splenocytes from AQP3-knockout mice than from wild-type mice after OVA challenge, consistent with findings showing that fewer CD4+ T cells from AQP3-knockout mice than from wild-type mice migrated to the lungs. Additionally, in vivo and in vitro experiments indicated that AQP3 induced the production of some chemokines, such as CCL24 and CCL22, by regulating the amount of cellular H_2_O_2_ in alternatively activated alveolar macrophages [70]. Because the pathological mechanisms of AR and asthma are generally similar, these results are expected to be similar in the AR model.

### 2.4. Relationship between AQPs and Chronic Rhinosinusitis with or without Nasal Polyp

Chronic rhinosinusitis (CRS) is a common upper respiratory tract inflammatory disease that occurs in the nasal cavity and sinus. It usually develops as a consequence of dysregulation of inflammatory response, owing to microbial infection in the nasal cavity and sinus, but various other pathological mechanisms are involved, as well. CRS has been divided into CRS with nasal polyps (CRSwNP), which has a tendency toward Th2 cytokine polarization, and CRS without nasal polyps (CRSsNP), which is associated with more of a Th1 type response [71]. Histologically, nasal polyps (NPs) are characterized by edematous fluid with sparse fibrous cells, few mucous glands with no innervation, squamous metaplasia of the surface epithelium, proliferation of stromal and epithelial elements, and thickening of the basement membrane [72]. It has been proposed that such immunological and histological differences are related to the differential expression of mucosal water membrane permeability proteins, such as AQPs. Diverse studies designed to demonstrate this are summarized in Table 3. 

A study by Altuntas et al. [56] comparing nasal polyp tissue and normal control tissue showed that AQP1 was expressed abundantly in polyp tissue fibroblasts, especially in the subepithelial area and the periphery of seromucous glands, as well as endothelial cells of venules. It is well-known that, in the early phase of inflammation, vasodilatation and increased blood flow increase intravascular hydrostatic pressure, resulting in increased filtration of fluid from the capillaries. Consequently, the permeability of the vessel wall increases, causing contraction of endothelial cells, widening of intercellular junctions or formation of intercellular gaps, and production of protein-rich exudates, resulting in edema [73]. However, this process only occurs within 30 min following exposure to the stimulus. Thus, the authors of this latter study suggested that elevation of AQP1 in NPs compared with normal tissue is among the mechanisms that contribute to chronic tissue edema in NPs. 

Shikani et al. compared differences in AQP5 expression between normal control, CRSwNP, and CRSsNP tissues, demonstrating that epithelial expression of AQP5 was significantly lower in CRSwNP, as compared with CRSsNP or controls. Thus, the authors suggested that the mucosal epithelial barrier is compromised in the context of CRS disease, especially CRSwNP, and that loss of AQP5, which might act as a tight junction protein, plays a role in the development of mucosal edema and the pathophysiology of nasal polyp formation [57]. Another study comparing normal control and CRSwNP tissue also reported a decrease in AQP5 in tissues from CRSwNP patients [58]. The authors of this study concluded that loss of AQP5 caused impaired tight junction regulation of water transport and cell volume and failure to maintain epithelial water homeostasis, resulting in edema and polyp formation and production of thick secretions—typical features of CRSwNP. 

Glucocorticoids are effective anti-inflammatory drugs for improving airway inflammatory diseases and are considered the first-line treatment for CRS [74]. Glucocorticoids are also reported to regulate water balance in various organs [75]. This latter effect was explored in a study that sought to elucidate the effectiveness of glucocorticoids in CRS by focusing on the inflammatory reaction and water homeostasis [59]. Using a rat model, the authors of this study compared expression levels of AQP5 between animals in control, CRS, and dexamethasone treatment groups. They found that expression of AQP5 was significantly increased in the dexamethasone-treated group, as compared with the other two groups. The authors postulated that the expression of AQP5 may be decreased in the CRS group because *Staphylococcus aureus*, the infectious microbe used in this study to elicit CRS, destroys the ciliated epithelium and glandular tissue in which AQP5 is primarily localized, but this latter effect is suppressed by dexamethasone, resulting in an increase in AQP5 expression. 

### 2.5. Potential Roles of AQPs in Nasal Inflammation

AR and CRS are the most common diseases related to respiratory function in the nose. The pathogenesis of these two diseases have numerous features in common, with edema of the nasal mucosa and hypersecretion being their typical pathological mechanisms. During this process, the roles of water channels like AQPs are inevitably significant. In previously highlighted studies, expression of AQP5, an AQP subtype found in respiratory epithelium of the normal nasal cavity, was shown to be decreased in AR and CRS. AQP5 expression, in this context, may be regulated by related transcriptional pathways, including NF-κB and cAMP-PKA/CREB pathways, and cytokines, such as IL-13 and TNF-α. Through their effects on AQP5 levels, these regulatory pathways are postulated to induce changes in intercellular tight junctions or gap junctions of nasal respiratory epithelial cells, resulting in edema—a common symptom of both diseases. Since drugs that possess anti-inflammatory effects, like dexamethasone and glycyrrhizin, act through these pathways to produce similar patterns of increased AQP5 expression, they could be anticipated to exert therapeutic effects by regulating the expression of AQPs in AR or CRS. However, no drug has been approved for this indication. In addition, because expression of AQPs in the respiratory epithelium of the nasal cavity changes in response to hyperosmotic stress, establishing the optimal concentration of NaCl solution used in nasal lavage may also be necessary.

## 3. Relationship between AQPs and Olfaction

The olfactory system is a unique sensory system in that olfactory sensory neurons are embedded in the epithelium in the nasal cavity and directly face the external environment. The olfactory mucosa is a yellowish mucous membrane located in the upper region of the nasal cavity [12,13]. Chemosensory epithelia, which are responsible for olfactory sensation, are categorized into two types: olfactory epithelium and vomeronasal sensory epithelium. The olfactory epithelium detects odors in the nasal cavity, whereas the vomeronasal organ (VNO) is more specifically involved in the detection of pheromonal substances released by congeners; however, most pheromones activate both vomeronasal and main olfactory sensory neurons, suggesting functional convergence of the two olfactory systems [76]. In both the olfactory epithelium and vomeronasal sensory epithelium, all cells that have access to the luminal surface via their apical surface, including chemosensory, supporting, and ductal cells, are involved in the chemosensing process. In these tissues, AQPs probably collaborate to regulate the water and ionic environment at the chemoreceptor surface, as well as in the intercellular space [12,13,76]. 

The olfactory epithelium is composed of olfactory sensory cells, supporting cells, and basal cells. The mucus protects the olfactory epithelium and allows odors to dissolve so that they can be detected by olfactory receptor neurons. The associated Bowman’s glands are present in the olfactory region, and their ducts penetrate the epithelium. Bowman’s glands, also known as olfactory glands, are a type of nasal gland situated in the olfactory mucosa beneath the olfactory epithelium in the lamina propria, a connective tissue that also contains fibroblasts, blood vessels, and bundles of fine axons from olfactory neurons. Bowman’s gland consists of an acinus in the lamina propria and a secretory duct that exits through the olfactory epithelium. Electron microscopy studies have shown that Bowman’s glands contain cells with large secretory vesicles [12,13]. The exact composition of the secretions from Bowman’s glands is unclear, but there is evidence that they produce odorant-binding proteins.

Water content is an important environmental factor in complex sensory organs such as the eye, ear, and nose, where maintenance of the specific environment surrounding sensory cells is crucial for proper function. In fact, multiple isoforms of AQPs have been found in these sensory organs. For example, AQP1–11 are expressed in the ear [77], and AQP0, -1, -2, -3, -4, -5, -6, -7, -9, and -11 are expressed in a tissue-specific manner in the eye [78]. Studies describing the expression of AQPs in nose as they pertain to olfaction are summarized in Table 4.

Immunohistochemical and immunoblot analyses of the rat nasal olfactory mucosa have detected the presence of AQP1, AQP3, AQP4, and AQP5 [16]. In this study, AQP1 expression in olfactory mucosae was found in endothelial cells of blood vessels and the surrounding connective tissue cells. However, AQP1 was not detected in olfactory sensory cells in the olfactory epithelium. Instead, AQP3 was abundant in the olfactory epithelium, where it was localized to supporting cells and basal cells; AQP4 expression in the olfactory epithelium was limited to basal cells. In Bowman’s gland, AQP5 was localized to apical membranes of secretory acinar cells, whereas AQP3 and AQP4 were found in the basolateral membrane. A similar localization pattern was seen in duct cells of Bowman’s gland. These results reveal a distinct localization pattern for AQPs in the olfactory epithelium. The authors suggested that AQP3 and AQP4 in supporting cells and basal cells may play an important role in generating and maintaining the specific microenvironment surrounding olfactory sensory cells. AQP3, -4, and -5 in Bowman’s gland may be involved in secretory processes that generate a microenvironment at the apical surface of olfactory dendrites suitable for odorant reception. A study in mice [79] showed that AQP1, -3, and -4 are present in the olfactory mucosa, giving rise to speculation that the AQP pathway and mucin act together to regulate homeostatic balance. In another study using rats [80], AQP4 was shown to be strongly expressed in the glomerulus—the synaptic unit of the olfactory bulb—but was largely undetectable in the olfactory nerve layer. Therefore, the authors suggested that AQP4 has some function in olfaction. In support of this, olfaction is reported to be diminished in AQP4-null mice [81]. 

The other chemosensory epithelium in the nasal cavity mucosa with an olfactory function is the vomeronasal sensory epithelium. As noted above, the VNO is part of the nasal chemosensory system that is anatomically and physiologically distinct from the olfactory system and is considered a chemosensory organ for pheromones. A rat study that investigated the expression of AQPs in VNO [82] found that AQP1 was localized to blood vessels and was particularly abundant in cavernous tissues of the non-sensory mucosa; AQP5 was detected in the apical membrane of gland acinar cells; AQP3 was detected in basal cells of the non-sensory epithelium, but it was absent in the sensory epithelium; and AQP4 was found in both sensory and non-sensory epithelia. Interestingly, AQP4 was highly concentrated in sensory cells of the sensory epithelium. An immunoelectron microscopic examination clearly showed that AQP4 is localized at the plasma membrane in the cell body and lateral membranes of dendrites, with the exception of the microvillous apical membrane. Nerve-fiber bundles emanating from neuronal sensory cells were found to express AQP4, such that the plasma membrane of each axon was positive for AQP4. These observations clearly show that neuronal sensory cells in the VMO are unique, in that they express abundant AQP4 at their plasma membranes. The authors noted that this pattern is in marked contrast to that in olfactory and central nervous systems, where AQPs are not detectable in neurons, and instead, AQP4 is abundant in supporting cells and the astrocytes surrounding them. Using an immunohistochemical approach in embryonic and postnatal mice, Mergio et al. [83] reported developmental stage-dependent expression of AQP2, -3, -4, and -5 in the olfactory epithelium and vomeronasal sensory epithelium. These authors showed that AQP2 is expressed in sustentacular cells of the VNO and olfactory epithelium, as well as in blood vessels of the underlying mucosa and VNO (but not Bowman’s glands); AQP4 is expressed in both chemosensory epithelia and in Bowman’s glands; AQP3 is expressed in the olfactory epithelium and associated Bowman’s glands, but not in the VNO chemosensory epithelium or glands; and AQP5 is expressed in the olfactory epithelium and both Bowman’s and VNO glands. The pattern of AQP expression in the olfactory epithelium differed between embryonic and postnatal periods, such that AQP2, AQP3, and AQP4 appeared at embryonic day (E) 14 and AQP5 appeared at E17. All AQPs except AQP2, which was no longer found in the olfactory epithelium after postnatal day (P) 15, persisted until adulthood. In the vomeronasal sensory epithelium, AQP2 appeared at E14 and was absent after P15, as was also observed in the olfactory epithelium; in contrast, AQP4 was expressed in the VNO sensory epithelium from E18 to P60, and AQP3 and AQP5 were absent at all developmental time points. In the respiratory epithelium, AQP2 and AQP4 were present from E14 to P60, whereas AQP3 and AQP5 appeared at E16 and P1, respectively. The patterns of AQP expression in olfactory and vomeronasal glands were different, with AQP3 and AQP4 being expressed in the former, AQP2 in the latter, and AQP5 in both. On the basis of the differential expression of AQPs according to rat developmental stage, the authors postulated that the regulation of water/ions would also differ according to the development of olfaction, which begins during the in utero period. Moreover, the authors suggested that the dissimilar distribution of AQPs in the olfactory epithelium and VNO, which detect odorants and pheromones, respectively, may also be one reason why the two organs exhibit different characteristics as they mature. 

From the olfactory epithelium, odorant-related information is carried via the olfactory nerve as it passes through the cribriform plate to the olfactory bulb. The olfactory nerve is composed not only of axons of olfactory receptor neurons but also of the cell bodies and extensive processes of olfactory ensheathing glia (OEG). This chemically unique type of glial cell, with characteristics of both astrocytes and Schwann cells, arises from the olfactory placodes, not the ventricular zone or neural crest, where other glial cells originate. As demonstrated in ultrastructural morphological studies, OEG envelop or surround large groups of axons of olfactory receptor neurons along their course within the lamina propria and into the olfactory nerve and glomerular layers of the olfactory bulb [84]. Extending these observations, researchers investigated the expression of AQP1 in OEG [85], examining the distribution of the AQP1 water channel in the olfactory bulb and lamina propria of embryonic and adult mice. They found that AQP1 is expressed in neurochemically identified OEG in the main olfactory bulb, but it is excluded from olfactory receptor neurons, astrocytes, and periglomerular cells. Expression of AQP1 is, therefore, a characteristic that distinguishes the OEG from other CNS cell types. 

## 4. Limitations of Studies Reported to Date and Future Research Plans

This review has summarized studies related to AQPs in nasal respiratory epithelium or olfactory epithelium. However, these studies had various limitations. The first is that most of these studies consisted of primary analysis of the expression or distribution of AQPs in nasal tissue. Although water flux in astrocytes was shown to change through surface trafficking/relocation without altering the level of expression of AQP4 [35,36], no studies to date in rhinology have reported changes or rearrangement of AQPs in cells. Therefore, further studies are needed to determine whether surface trafficking/relocation of AQPs is similar in nasal tissue cells and whether this affects water flux. The second limitation is that most studies to date have been performed in primary cultured cells. To our knowledge, no studies have assessed the relationships between the distribution of various types of AQPs in the nasal cavity and their specific function or mechanism of action. Although in vitro and in vivo results may be similar, they may also be completely different, emphasizing the importance of in vivo studies. Although many studies have evaluated AQPs in rhinology, these results are insufficient to determine their pathophysiological mechanisms. Therefore, in vivo studies are needed to determine the roles and mechanisms of action of AQPs in rhinology.

## Figures and Tables

**Table 1:** Studies assessing the expression of aquaporins (AQPs) in normal respiratory epithelium of the nasal cavity.

Authors and Reference	Study Design	Species and/or Tissue Type	Detection Method	Target AQPs	Results/Conclusion
Nielsen et al. [14]	Animal tissue study	Rats; respiratory tract, glandular tissues	Immunocyto-chemistry, immunoelectron microscopy	Expression and localization of AQP1, -3, -4, -5	AQP1: nasopharyngeal vascular endothelium and fibroblasts AQP5: apical plasma membranes of secretory epithelium in upper airway and salivary glands AQP3: basal cells of nasopharyngeal epithelium, basolateral membranes of surface epithelial cells of the nasal conchus AQP4: nasopharyngeal epithelium, nasal conchus
Ablimit et al. [15]	Animal tissue study	Rats; nasal olfactory and respiratory mucosae)	Immunocyto-chemistry, immunoblotting	Expression and localization of AQP1, -3, -4, -5	AQP1: endothelial cells of blood vessels and surrounding connective tissue cells AQP3: mostly restricted to basal cells, relatively weak expression in ciliated columnar cells AQP4: abundant in cells of the respiratory epithelium, such as ciliated columnar cells and basal cells AQP5: apical side of glandular cells
Sakai et al. [16]	Animal tissue study	Mice	qRT-PCR	Expression of AQP0, -1, -2, -3, -4, -5, -6, -7, -8, -9, -11, -12	The genes for AQP1, -3, -4, -5, and -11 were notably expressed in the nasal epithelium.
Maeda et al. [17]	Animal tissue study	Musk shrew (*Suncus murinus*), rat	Immunohisto-chemistry, immunoblotting	Expression of AQP1, -3, -4, -5	Musk shrew: AQP1, -3, -4, -5 was observed but only a small amount of AQP3, -4, -5 was found. Rat: AQP1, -3, -4, -5 are observed, and AQP3,4 is particularly abundant.
Vesterdorfet al. [18]	Animal tissue study	Sheep (*Ovis aries*)	Immunohisto-chemistry	AQP1, -3, -5	AQP1: connective tissue, vascular endothelial cells, glandular acini AQP3: ciliated columnar epithelial cells (basolaterally), basal cells AQP5: glandular acini (apically), glandular duct cells, ciliated columnar epithelial cells (apically)
Seno et al. [19]	Human tissue study	Human (normal control vs. AR vs. CRSwNP)	RT-PCR, immunoblotting, immunohistochemistry	AQP1, -2, -3, -4, -5	AQP1: fibroblasts (especially in the subepithelial area), endothelial cells of blood vessels AQP2: cytoplasm of epithelial cells and acinar cells AQP3: basolateral sites of epithelial cells and acinar cells AQP4: basolateral sites of acinar cells AQP5: apical sites of epithelial cells and acinar cells
Frauen-felder et al. [20]	Human tissue study	Human (normal control vs. CRSwNP vs. CRSsNP)	RT-PCR, immunohistochemistry	AQP0, -1, -2, -3, -4, -5, -6, -7, -8, -9, -10, -11, -12b	AQP1: vasculature and connective tissue AQP3: basolateral membranes of both surface and glandular epithelium > apical membrane and cell cytoplasm AQP4: apical membrane of the surface epithelium, connective tissue AQP5: surface epithelium (apical and basolateral membranes > cytoplasm), glandular epithelium (apical membranes > basolateral membranes and cytoplasm) AQP7 and 11: cytoplasm of the surface and glandular epithelium
Matsuzaki et al. [21]	Animal tissue study	Rats	Immunoblotting, immuno-fluorescence microscopy, immunoelectron microscopy	AQP3	AQP3: pseudostratified ciliated epithelium in the nasal cavity

AQP, aquaporin; qRT-PCR, quantitative reverse transcription polymerase chain reaction; AR, allergic rhinitis; CRSwNP, chronic rhinosinusitis with nasal polyp; CRSsNP, chronic rhinosinusitis without nasal polyp.

**Table 2:** Studies assessing the factors that regulate the expression of AQPs in the respiratory epithelium of the nasal cavity.

Authors and Reference	Study Design	Species and/or Tissue Type	Detection Method	Associated Factors	Target AQPs	Results/Conclusion
Müller et al. [43]	Animal tissue study (case-control)	Duckling (*Anas platyrhynchos*)	RT-PCR, northern blotting, immunoblotting, immunohistochemistry	Osmotic stress (hyperosmotic fluid)	AQP1, -5	AQP1 and AQP5 expression were decreased during saline adaptation (replacement of drinking water with a 1% NaCl solution) in ducklings.
Pedersen et al. [44]	In vitro- cultured cell study	Primary cultured HNEpCs	Immunofluorescence, confocal microscopy	Osmotic stress (hyperosmotic fluid)	AQP5	Hyperosmotic stress is an important activator of AQP5 in the human nasal epithelium, and leads to significantly increased transepithelial water permeability.
Huang et al. [45]	In vitro- cultured cell study	Primary cultured HNEpCs	Immunohistochemistry, immunoblotting	Treated with chitosan	AQP3, -5	Chitosan treatment significantly increased AQP3 and AQP5 expression in HNEpCs compared with control groups.
Wang et al. [46]	Animal tissue study	Rats	RT-PCR, immunoblotting, immunohistochemistry	cAMP-PKA/CREB pathway	AQP5	AQP5 expression was decreased by inhibition of PKA (H89) and increased by stimulation of adenylyl cyclase-dependent cAMP production (forskolin).
Jun et al. [47]	In vitro-cultured cell study	Primary cultured HNEpCs	RT-PCR	Various culture conditions (permeable filter vs. dish)	AQP3, -4	Culture of HNEpCs on permeable filters induced expression of AQP3 and AQP4.

AQP, aquaporin; qRT-PCR: quantitative reverse transcription polymerase chain reaction; HNEpCs, human nasal epithelial cells; cAMP-PKA/CREB, cyclic adenosine monophosphate-protein kinase A/cyclic adenosine monophosphate response element-binding protein.

**Table 3:** Studies assessing the relationship between AQPs and inflammatory diseases in the nasal cavity.

Authors and Reference	Study Design	Species and/or Tissue Type	Detection Method	Associated Diseases	Target AQPs	Results/Conclusion
Seno et al. [19]	Human tissue study	Human (normal control vs. AR vs. CRSwNP)	RT-PCR, immunoblotting, immunohistochemistry	AR, CRSwNP	AQP1, -2, -3, -4, -5	There were no significant differences in expression of AQP1, AQP3, or AQP5 mRNAs among control, AR, and CRSwNP patients.
Frauenfelder et al. [20]	Human tissue study	Human (normal control vs. CRSwNP vs. CRSsNP)	RT-PCR, immunohistochemistry	CRSwNP, CRSsNP	AQP0, -1, -2, -3, -4, -5, -6, -7, -8, -9, -10, -11, -12b	AQP4 and AQP11 mRNA expression patterns were significantly different in CRSwNP, and localization patterns of AQP4 and AQP5 protein were different in both types of CRS.
Wang et al. [50]	Animal tissue study	Rats	RT-PCR, immunoblotting, immunohistochemistry	AR (induced by aluminum)	AQP5	IL-1β acts through the NF-κB pathway to downregulate AQP5 by inhibiting CREB phosphorylation, or through NF-κBp65, which competitively bound CBP.
Chang et al. [51]	In vitro cultured cell study	Primary cultured HNEpCs	Immunoblotting, immunohistochemistry	AR (histamine, chlorpheniramine)	AQP5	Chlorpheniramine attenuates histamine-induced downregulation of AQP5 in HNEpCs via suppression of NF-κB activation.
Wang et al. [52]	In vitro cell line study	HNEpCs	RT-PCR, northern blotting, immunoblotting, immunohistochemistry	AR (induced by histamine)	AQP5	Histamine downregulates AQP5 production in HNEpCs by inhibiting CREB phosphorylation at Ser-133.
Li et al. [53]	In vitro cell line study	HNEpCs	RT-PCR, immunoblotting, ELISA	AR (histamine, glycyrrhizin)	AQP5	Expression of AQP5 in HNEpCs was decreased by histamine and increased by glycyrrhizin.
Chang et al. [54]	In vitro cultured cell study	Primary cultured HNEpCs	Immunoblotting, immunohistochemistry	AR (methacholine, dexamethasone)	AQP5	Dexamethasone attenuates methacholine-induced downregulation of AQP5 in HNEpCs via suppression of NF-κB activation.
Skowron-zwarg et al. [55]	In vitro-cultured cell study	primary cultured HNEpCs	RT-PCR, immunofluorescence, immunohistochemistry, immunoblotting	AR (IL-13)	AQP3, -4, -5	IL-13 did not affect AQP3 or AQP4 expression, but did abolish AQP5 expression.
Altuntas et al. [56]	Human tissue study	Human (normal control vs. CRSwNP)	RT-PCR, immunoblotting, immunohistochemistry	CRSwNP	AQP1, -4	AQP1 was highly expressed in fibroblasts located in polyp tissue, especially in the subepithelial area and the periphery of seromucous glands, as well as in endothelial cells of venules.
Shikani et al. [57]	Human tissue study	Human (normal control vs. CRSwNP vs. CRSsNP)	RT-PCR, immunoblotting, immunohistochemistry	CRSwNP, CRSsNP	AQP5	The epithelial expression of AQP5 was significantly lower in CRSwNP compared with CRSsNP or controls.
Pistochini et al. [58]	Human tissue study	Human (normal control vs. CRSwNP)	RT-PCR	CRSwNP	AQP5	*AQP5* gene was significantly downregulated in CRSwNP samples compared with those from control groups.
Yu et al. [59]	Animal tissue study	Rats (normal control vs. CRS vs. CRS treated with dexamethasone)	RT-PCR, immunohistochemistry	CRS (dexamethasone)	AQP5	AQP5 mRNA expression level was significantly higher in the dexamethasone group than in control and CRS groups.

AQP, aquaporin; qRT-PCR, quantitative reverse transcription polymerase chain reaction; AR, allergic rhinitis; CRSwNP, chronic rhinosinusitis with nasal polyp; HNEpCs, human nasal epithelial cells; CREB, cyclic adenosine monophosphate-responsive element binding protein; NF-κB, nuclear factor kappa B; IL, interleukin; CBP, CREB-binding protein; ELISA, enzyme-linked immunosorbent assay; CRSsNP, chronic rhinosinusitis without nasal polyp.

**Table 4:** Studies assessing the relationship between AQPs and olfaction.

Authors and Reference	Study Design	Species and/or Tissue Type	Detection Method	Target AQPs	Results/Conclusion
Solbu et al. [56]	Animal tissue study	Rats and mice (olfactory mucosa)	Immunofluorescence staining, immunogold electron microscopy	AQP1, -3, -4, -5	AQP5 is present at the apical surface of the olfactory epithelium, and an intricate network of fine AQP1-positive fibroblast processes surrounds Bowman’s glands.
Sørbø et al. [73]	Animal tissue study	Rats (nasal epithelium and glomerulus of the olfactory bulb)	RT-PCR, immunoblotting, immunohistochemistry	AQP4	AQP4 is strongly expressed in the glomerulus, the synaptic unit of the olfactory bulb, suggesting a role for AQP4 in olfactory function.
Lu et al. [57]	Animal tissue study	Mice (AQP4-null mice vs. wild-type mice)	Immunocytochemistry, immunofluorescence	AQP4	The olfactory epithelium of AQP4-null mice appeared outwardly normal, but did not express AQP4, exhibited a 12-fold reduction in osmotic water permeability, and showed impaired olfaction.
Ablimit et al. [58]	Animal tissue study	Rats (VNO)	Immunofluorescence microscopy, immunoelectron microscopy	AQP1, -2, -3, -4, -5, -6, -7, -9, -10, -11	The localization of AQPs in the VNO is distinct from that in the olfactory mucosa. AQP4 was specifically detected in neuronal sensory cells, which is also a unique feature among neuronal cells.
Merigo et al. [74]	Animal tissue study	Mice (VNO vs. olfactory mucosa)	RT-PCR, immunoblotting, ELISA	AQP2, -3, -4, -5	In the olfactory epithelium, AQP2, AQP3, and AQP4 appeared at E14 and AQP5 at E17, and all persisted until adulthood except AQP2, which was no longer found in olfactory epithelium after P15. In the VNO–SE, AQP2 appeared at E14 and was absent after P15, as was also observed in the olfactory epithelium; instead, AQP4 was expressed in the VNO–SE from E18 to P60, and AQP3 and AQP5 were absent throughout.
Shields et al. [59]	In vivo animal study, in vitro cultured cell study	Mice	Immunoblotting, immunohistochemistry	AQP1	AQP1 is expressed in lamina propria of the nasal cavity, OEG in the main olfactory bulb, and in primary cultured olfactory bulb cells.

AQP, aquaporin; qRT-PCR, quantitative reverse transcription polymerase chain reaction; VNO, vomeronasal organ; E, embryonic day; P, postnatal day; VNO–SE: vomeronasal–sensory epithelium; OEG, olfactory ensheathing glia.
